# Knowledge and Misconceptions About Antibiotic Use and Resistance Among Dental Clinic Visitors in Saudi Arabia—A Cross-Sectional Study

**DOI:** 10.3390/healthcare13161971

**Published:** 2025-08-11

**Authors:** Sarah R. Alharbi, Lama Alzamil, Zeina S. Alkudmani, Amal Alhani, Layla Faqih, Esraa Aldawood

**Affiliations:** 1 Department of Clinical Laboratory Sciences, College of Applied Medical Sciences, King Saud University, P.O. Box 145111, Riyadh 12372, Saudi Arabia; lalzamil@ksu.edu.sa (L.A.); zalkudmani@ksu.edu.sa (Z.S.A.); lfaqih@ksu.edu.sa (L.F.); ealdawood@ksu.edu.sa (E.A.); 2 Department of Public Health, College of Veterinary Medicine, Cornell University, Ithaca, NY 14853, USA; aa2577@cornell.edu

**Keywords:** antibiotics, antibiotic resistance, antibiotic misuse, antibiotic stewardship, public awareness, dental patients, dentistry, Saudi Arabia

## Abstract

**Background/Objectives**: Antibiotic resistance (AR) is a major public health concern, mainly driven by the misuse of antibiotics. Dental settings are one area where unnecessary antibiotic prescriptions and misconceptions among dental patients contribute significantly to antibiotic misuse. This study aimed to evaluate the knowledge and understanding of antibiotic use and AR among dental clinic visitors in Saudi Arabia. **Methods**: A cross-sectional survey was conducted between March and June 2024, targeting males and females aged 18 years and older residing in Saudi Arabia who visited a dental clinic within the past five years. A self-administered questionnaire was used to assess participants’ knowledge of antibiotic use and their awareness of AR. **Results**: A total of 1455 respondents from different regions of Saudi Arabia completed the survey. The majority of participants (70.1%) correctly identified that antibiotics kill bacteria, but around 30% incorrectly believed antibiotics treat viral infections. Additionally, 19.58% thought it was acceptable to stop taking antibiotics once they feel better. More than half of the participants thought that antibiotics reduce inflammation (69.10%) or relieve pain (52.40%). Nearly half of the participants (48.45%) were unaware of the term “antibiotic resistance (AR),” and only 27.2% identified antibiotic overuse as a cause of AR. A significant association was found between undergoing dental procedures and perceiving antibiotics as necessary (*p* < 0.0001). Educational level was the only significant predictor of AR awareness (AOR = 2.942; *p* < 0.0001). **Conclusions**: Although many participants correctly answered a knowledge question about antibiotics killing bacteria, several misconceptions about antibiotic use and a lack of awareness regarding AR were identified. These findings highlight the need for targeted educational interventions and enhanced antibiotic stewardship in dental practice to control AR.

## 1. Introduction

Antimicrobial resistance (AMR) is one of the most pressing global public health threats, affecting people, animals, and the environment [1,2]. According to a landmark study published in The Lancet, bacterial AMR was associated with 4.95 million deaths in 2019, including 1.27 million deaths directly caused by resistant bacteria [1].

Several factors contribute to the rise in bacterial AMR, including the overuse and misuse of antibiotics in both human medicine and agriculture [3]. In response, the World Health Organization (WHO) has launched a Global Action Plan to improve awareness, strengthen surveillance, and promote the responsible use of antimicrobials [4]. Misconceptions about the indication of antibiotics are common worldwide among the public, often leading to antibiotic misuse. Several international surveys, including the WHO multi-country public awareness survey and the Public Health England survey, reported common misconceptions such as the belief that antibiotics are effective against viral infections, as well as misuse behaviors, including not completing the prescribed course of antibiotics and self-medicating with leftover antibiotics [5,6,7]. Similar misconceptions and antibiotic misuse behaviors were also reported among the public in Saudi Arabia [8,9,10,11].

Antibiotics play a crucial role in managing bacterial infections in dentistry and as prophylaxis in certain cases [12]. However, despite evidence-based guidelines, such as those published by the American Association of Endodontists (AAE) regarding the proper use of antibiotics in treating oral infections, studies reported that dentists still prescribe antibiotics inappropriately and unnecessarily [13,14]. Another concern in dental care is the misuse of antibiotics through self-medication among patients. Many individuals obtain antibiotics without a prescription, believing they can treat dental pain or infections [15]. A 2024 systematic review, which included data from multiple low- and middle-income countries, found that the overall estimated prevalence of self-medication was 59% among individuals and 58% among dental patients. Approximately 19% of the drugs used without prescription were antibiotics, while about 60% were analgesics [16]. This antibiotic misuse can lead to serious consequences, including treatment failure, the development of antibiotic resistance (AR), and severe drug reactions [15].

In Saudi Arabia, several studies investigated antibiotic prescription practices and knowledge among dental professionals and dental students [17,18,19,20,21]. Alhobeira et al. found that despite moderate knowledge, a substantial percentage of dentists in the Hail region continued to prescribe antibiotics indiscriminately, emphasizing the need for enhanced education and prescribing guidelines [18]. Another study evaluated the knowledge and attitudes regarding antibiotic prescription among dental interns and dental students in their final clinical years (4th, 5th, and 6th years) at multiple universities in Saudi Arabia. The study highlighted that senior dental students (in their 6th year) and interns had a better understanding and more positive attitudes towards antibiotic prescription for oral infections compared to students in earlier academic years. However, the study also reported that all participating students and interns might inappropriately prescribe antibiotics for systemic conditions [19].

Despite growing attention to antibiotic awareness among dental professionals and students, studies specifically assessing the knowledge of antibiotics among dental patients in Saudi Arabia appear to be limited. To our knowledge, only one recent study provides relevant insights. This study evaluated dental patients’ adherence to prescribed antibiotics and their awareness of AMR. The findings of this study reported a high rate of non-adherence to antibiotic therapy, with 62.3% demonstrating low adherence, 34% showing moderate adherence, and only 3.6% showing complete adherence [22].

The present study is one of the first to investigate the level of knowledge and awareness about antibiotic use and AR among the public in dental settings. We aimed to assess the knowledge of dental clinic visitors in Saudi Arabia regarding antibiotic use, their understanding of AR and its causes, as well as their perception of the necessity of antibiotics after dental procedures.

## 2. Materials and Methods

### 2.1. Study Design and Setting

We conducted a cross-sectional survey targeting males and females aged 18 years and older residing in Saudi Arabia who visited a dental clinic within the past five years. The required minimum sample size was calculated to be 377 participants using Raosoft, Inc. (Seattle, WA, USA) “(http://www.raosoft.com/samplesize.html) (accessed on 15 May 2023)”, with a 5% margin of error and 95% confidence level. However, the final sample size was increased to 1455 participants to account for baseline variability in participants’ demographics (e.g., region, gender, education) and to reduce potential sampling errors. The final sample of 1455 reflects all complete and eligible responses collected during the study.

### 2.2. Questionnaire

The questionnaire (Supplementary File S1) began with two screening questions to confirm that participants were aged 18 years and older and visited a dental clinic in the past five years (questions 1 and 2). The remaining questions (questions 3–16) were organized into four sections. The first section collected sociodemographic information, including age, gender, nationality, educational level, and region of residence in Saudi Arabia (questions 3–7). The second section assessed participants’ knowledge of antibiotic use (questions 8–10). The third section assessed participants’ views on the necessity of antibiotics after dental procedures (questions 11 and 12). The fourth section evaluated awareness of AR, sources of information, and beliefs regarding its causes and severity (questions 13–16).

The survey questions 1 and 2 were developed by the authors. Questions 3, 4, 5, 6, 7, and 9–13 were adapted from [23].The adaptation included adding questions 5 and 6 to collect data about participants’ city of residence and nationality, expanding question 7 to include a detailed education scale (no studies, primary school, diploma, postgraduate degree, and doctorate degree (PhD) and modifying questions 11 and 12 to include multiple dental procedures (teeth extraction, root canal treatment, and gum surgery). Domínguez-Domínguez et al. implemented a similar adaptation approach to questions 7, 11, and 12 [24]. Question 13, which was also adapted from the WHO survey (see below), was modified to “Are you aware of the term ‘antibiotic resistance’?”

Questions 8 and 13 −16 were adapted from the WHO multi-country public awareness survey [5] as follows: questions 8 and 15 were structured based on the key findings and behavioral items from the WHO survey, including antibiotic sharing, non-adherence, and misconceptions about antibiotic treatment for colds, flu, and other viral infections. For question 14, the option “school curriculum” was added as a possible source of information. Question 16 was adapted from a multi-statement item in the WHO survey assessing public perception of the seriousness of the antibiotic resistance issue. This was simplified into a single direct question (Q16): “Do you believe antibiotic resistance is a serious problem?” with yes/no response options.

The questionnaire was initially developed in English and then translated into Arabic by the first author (S.R.A). A professional linguist subsequently reviewed it for linguistic accuracy and cultural appropriateness. Both the English and Arabic versions of the questions were presented side by side in the questionnaire in a bilingual format, allowing participants to choose their preferred language. The final review of the questionnaire was conducted by S.R.A., E.A, and two dentists, the co-author A.A., and a dental researcher from the College of Dentistry at King Saud University. A pilot study with 30 participants was conducted to assess the clarity of the questions and the time required for completion; however, its results were not included in the final analysis.

### 2.3. Data Collection

After obtaining ethical approval, a self-administered online survey was created using Microsoft Forms. The survey link was distributed across various regions in Saudi Arabia through multiple social media platforms, such as WhatsApp and Telegram. Data were collected over a four-month period, from March to June 2024.

### 2.4. Ethical Consideration

Participants were informed about the study’s purpose and voluntarily completed the survey after providing informed consent. All responses remained anonymous and confidential. The study received ethical approval from the Institutional Review Board at KSU (Ref. No. 23/0515/IRB). Data confidentiality was strictly maintained and accessible only to the research team.

### 2.5. Statistical Analysis

Categorical variables were calculated using percentages. A logistic regression model was applied to examine the association between awareness of AR and sociodemographic factors, with awareness (yes vs. no) as the dependent variable. Independent variables included age, gender, nationality, educational level, and region of residence in Saudi Arabia. Adjusted odds ratios (AORs) and 95% confidence intervals (CIs) were reported. All statistical analyses were performed using GraphPad Prism version 10 (GraphPad Software, San Diego, CA, USA) and Microsoft Excel (Microsoft Corporation, Redmond, WA, USA).

## 3. Results

### 3.1. Demographic Characteristics of Respondents

In the current study, 1455 participants completed the survey (Table 1). The majority were female (60.07%). The age distribution showed that 43.71% of respondents were 18–25 years old, followed by 21.24% aged 26–35 years, with a very small number of seniors (60+ years) participating in the study. Almost all participants (96.01%) were Saudi. Educational levels varied among participants, with more than half (62.27%) holding a bachelor’s degree, while 15% had a high school diploma or less. Diploma and postgraduate holders comprised approximately 11% and 10%, respectively. Participants were distributed geographically across Saudi Arabia, with most residing in the central region (59.45%).

### 3.2. Knowledge of Antibiotic Use

**Figure 1:**
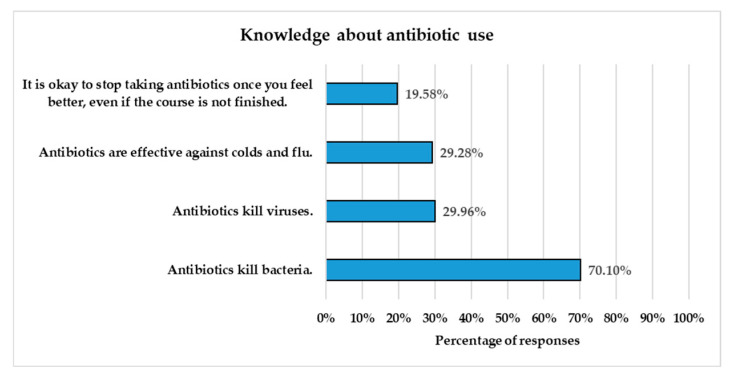
Respondents’ knowledge about antibiotic use. The *Y*-axis presents four statements reflecting common facts and misconceptions about antibiotics, while the *X*-axis shows the percentage of respondents who agreed with each statement (*n* = 1455).

Most respondents (70.10%) correctly identified that antibiotics kill bacteria (Figure 1). However, significant misconceptions were observed: around 30% of respondents believed that antibiotics kill viruses, 29.28% thought antibiotics are effective against colds and flu, and 19.58% believed it is acceptable to stop taking antibiotics once they feel better, even if the prescribed course is not completed.

### 3.3. Perceived Benefits and Adverse Effects of Antibiotics

**Figure 2:**
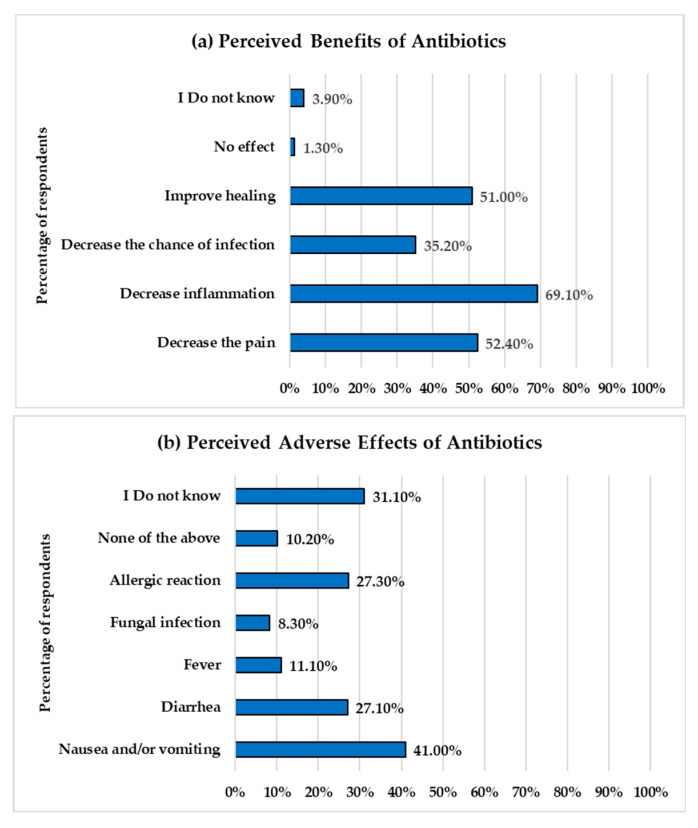
Perceived benefits and adverse effects of antibiotics among respondents. The *Y*-axes show the response options related to perceived benefits (**a**) and adverse effects (**b**) of antibiotics. The *X*-axes represent the percentage of respondents who selected each response.

Participants were asked to identify what they considered the benefits and adverse effects of antibiotics, with the option to select more than one response for each. Figure 2 illustrates the participants’ perceptions of the benefits and adverse effects of antibiotics.

The results reveal misconceptions regarding the benefits of antibiotics. The majority of respondents, 69.10% (1006 out of 1455), believed that antibiotics reduce inflammation. Approximately half of them indicated that antibiotics decrease pain and improve healing. About one-third (35%) of participants acknowledged that antibiotics decrease the chance of infection, which is a correct benefit of antibiotics. Only 3.9% of participants were unaware of the benefits of antibiotics, and 1.3% believed that antibiotics have no benefit.

The most commonly reported adverse effects of antibiotics were nausea and/or vomiting (597 responses, 41%), followed by allergic reactions (27.30%) and diarrhea (27.1%). A small proportion identified fever (11.10%) and fungal infections (8.30%). A significant proportion (31%) of participants were unaware of the side effects of antibiotics.

### 3.4. Perception of the Necessity of Antibiotics After Dental Procedures

**Figure 3:**
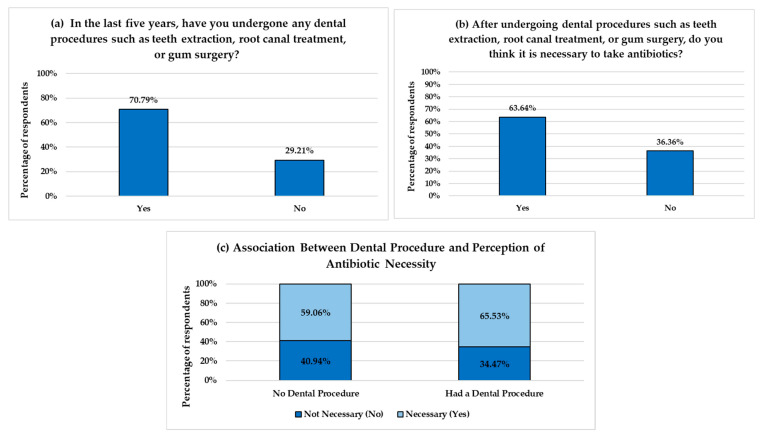
Perception on the necessity of antibiotics after dental procedures. (**a**,**b**) show respondents’ answers regarding their history of undergoing dental procedures and their beliefs in the necessity of antibiotics following such procedures. (**c**) illustrates the relationship between undergoing a dental procedure and the perception that antibiotics are necessary. The *Y*-axes represent the percentage of respondents, and the *X*-axes represent response categories.

Participants’ perceptions regarding the necessity of antibiotics following dental procedures were evaluated (Figure 3). The majority of respondents, 70.79% (*n* = 1030), reported that they underwent a dental procedure such as tooth extractions, root canals, or gum surgery within the past five years. Among all respondents (*n* = 1455), 63.36% believed antibiotics were necessary following such dental procedures. To determine whether past dental experiences influenced this perception, responses were compared between participants who underwent dental procedures in the past five years and those who had not. The results showed that 65.53% of participants with a history of dental procedures believed antibiotics were necessary, compared to 59.06% of those without such experience (Figure 3c). A Chi-square test revealed a statistically significant association between having undergone dental procedures and the perception of antibiotic necessity (χ^2^ = 83.63, df = 1, *p* < 0.0001).

### 3.5. Awareness of Antibiotic Resistance

**Figure 4:**
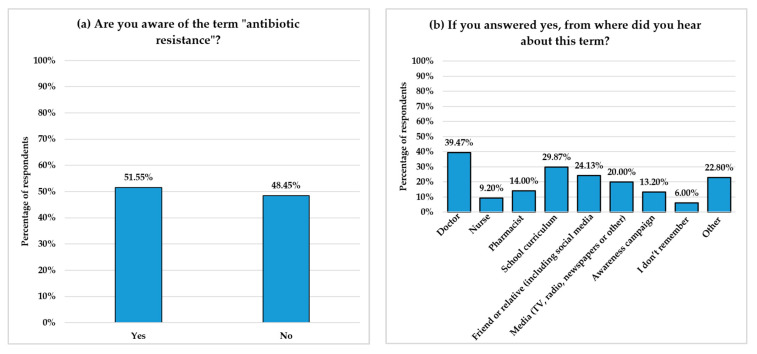
Awareness and sources of information about AR among respondents. (**a**) shows the percentage of responses to the question, “Are you aware of the term ‘antibiotic resistance‘?” (**b**) illustrates the sources through which respondents who were aware of the term received information. The *Y*-axes represent the percentage of respondents, while the *X*-axes indicate response options.

Of the 1455 respondents, 705 (48.45%) were not aware of the term “antibiotic resistance,” while 750 (51.55%) reported that they were aware of the term (Figure 4a). The most frequently reported source of information among those who were aware was doctors (39.47%), followed by school curricula (29.87%) and friends or relatives (24.13%) (Figure 4b). Notably, pharmacists and nurses were the least common sources of information, reported by 14% and 9.20%, respectively.

**Figure 5:**
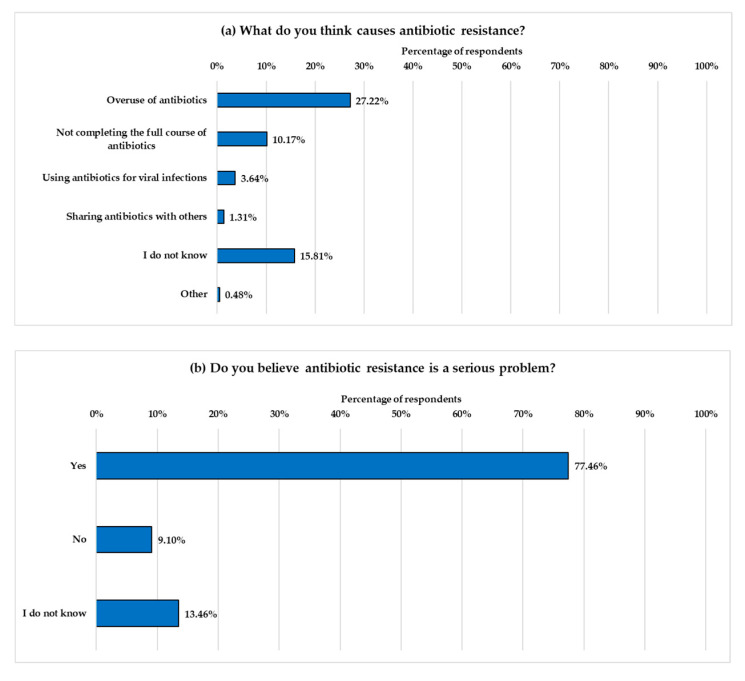
Participants’ beliefs about the causes and seriousness of AR. (**a**) presents participants’ perceptions of factors contributing to AR, while (**b**) shows participants’ responses to whether they view AR as a serious public health issue. The *Y*-axes display the response options, and the *X*-axes represent the percentage of participants selecting each option.

Participants who were aware of the term “antibiotic resistance” were asked about its causes and their perceptions of its severity (Figure 5). The most frequently identified cause was the overuse of antibiotics (27.22%) (Figure 5). Only a small number of respondents recognized other causes of AR. Alarmingly, 15.81% stated they were unsure about the causes of AR. When asked, “Do you believe antibiotic resistance is a serious problem?”, the majority (77.46%) correctly replied “yes,” indicating general concern among those familiar with the term. However, 101 participants (13.47%) responded, “I do not know,” and 68 participants (9.07%) replied “no.”

### 3.6. Prediction of Antibiotic Resistance Awareness

A multivariate logistic regression analysis was conducted to predict awareness of AR based on participants’ sociodemographic characteristics (Table 2). Of the 1455 respondents, 24 had missing values in one or more covariates required for multivariable analysis and were excluded. The analysis was therefore conducted on 1431 complete cases. The analysis revealed that educational level was the only significant predictor of AR. Participants with a postgraduate degree were significantly more likely to be aware of AR (AOR = 2.942; 95% CI: 1.988–4.428; *p* < 0.0001). No significant associations were found between awareness and other variables, including age, gender, nationality, or region of residence.

## 4. Discussion

This study highlights both strengths in fundamental knowledge and critical gaps in understanding and awareness of antibiotic use and AR among dental clinic visitors. The majority of participants (71.10%) correctly identified that antibiotics kill bacteria. This finding aligns with previous local studies targeting the general public, which reported a good understanding of the activity of antibiotics against bacterial infections among the majority of participants [9,11,25]. However, misconceptions were observed in our study, particularly regarding the effectiveness of antibiotics against viruses and viral infections, such as the common cold and flu, as well as the importance of completing an antibiotic course.

Overall, the rate of misconceptions reported in our study is lower than that reported in other local and international studies targeting the general public. In this study, approximately 30% of respondents incorrectly believed that antibiotics can kill viruses and are effective against colds and flu. However, higher rates of misconception, ranging from 47% to 60%, were reported among the general public in Saudi Arabia [8,9,10,25]. Previous international surveys also reported higher misconception levels. According to a WHO survey conducted in 12 countries to assess public awareness and behaviors related to antibiotics, 64% of the participants mistakenly believed that antibiotics are effective against viral infections, such as the common cold or flu [5]. Similarly, Public Health England reported that 35% of the UK public believed antibiotics are used to treat viruses [6].

Furthermore, our study found that 19.58% of respondents believed it was acceptable to stop taking antibiotics once they felt better. This misconception is widespread globally. The WHO multi-country public awareness survey found that 32% of participants did not understand the importance of completing their prescribed course of antibiotics and believed they could stop taking them once they felt better [5]. Although our findings may not directly reflect participants’ actual adherence to antibiotic therapy, they highlight a potential risk for non-adherence behavior. Lack of antibiotic adherence exposes pathogenic bacteria to sub-lethal concentrations of antibiotics, allowing them to survive and develop AR, which can lead to treatment failure and the spread of resistant bacteria [26]. High non-adherence rates among the public were reported in several local studies. A recent study on dental patients across several regions in Saudi Arabia found that 68.8% of participants reported stopping their antibiotic treatment when they felt better [22]. Similarly, other local studies reported that early discontinuation of antibiotics is a common practice among the general public [25,27,28].

Proper knowledge of antibiotics’ benefits and adverse effects is crucial for preventing antibiotic misuse practices and the spread of AR. In our study, the most frequently selected benefits of antibiotics by participants are the reduction of inflammation and pain, both of which are incorrect perceptions regarding antibiotics [29]. Antibiotics do not directly reduce inflammation; however, reducing inflammation and pain are secondary effects that occur when the infection is treated. These misconceptions were also reported in previous studies among dental patients and the general public, suggesting a common perception among the public that antibiotics are medications with analgesic or anti-inflammatory properties [23,24,30,31,32,33].

A percentage of participants were aware of the common adverse effects of antibiotics, such as nausea and vomiting (40%), and diarrhea and allergic reactions (around 27%), which are well-recognized and clinically documented side effects of antibiotic use [34]. However, the level of uncertainty regarding adverse effects was higher (31%) than the uncertainty about the benefits of antibiotics (3.9%). This suggests that participants have a strong perception of the benefits of antibiotics but limited concerns about their potential harm, which may contribute to the misuse of antibiotics.

Our study identified a significant association between undergoing a dental procedure (e.g., teeth extraction and root canal treatment) and the perception that antibiotics are necessary. Our results align with previous studies, which report that dental patients often consider antibiotics after tooth extraction or root canal treatment [23,24]. Patients’ past clinical experiences, such as receiving antibiotics without explanation from their healthcare provider, being prescribed antibiotics unnecessarily, or receiving them for prophylactic purposes, significantly influence their beliefs about the necessity of antibiotics [35]. In Saudi Arabia, studies reported unnecessary antibiotic prescriptions by dentists for dental conditions like symptomatic apical periodontitis (SAP), which often require only local treatment [36,37]. Also, studies showed that patients experiencing dental pain often receive antibiotics as an urgent management strategy without applying local dental treatment, which may explain why they associate antibiotics with pain relief [33,38]. These experiences can lead to an unnecessary demand for antibiotics, which can place pressure on dentists’ prescribing practices [39,40]

Understanding public awareness and knowledge of AR and identifying the sources of this knowledge is essential for designing effective educational interventions [41,42]. The present study revealed that nearly half of the respondents (48.45%) were unaware of the term “antibiotic resistance”. Among those aware of the term, the most common sources of information identified were doctors, followed by the school curriculum. Although pharmacists and nurses play critical roles in healthcare settings, they were less frequently identified by respondents as sources of AR knowledge. Similarly, public health campaigns, which play a crucial role in improving awareness of AR and promoting the appropriate use of antibiotics [43], were identified by only 99 respondents (13.2%). This finding suggests the limited engagement of current local AMR campaigns, despite recommendations from the Saudi AMR action plan to enhance public awareness about AMR [42]

Among participants aware of the term “antibiotic resistance,” only 27.22% recognized that antibiotic overuse contributes to the development of resistance. The number of respondents who identified other significant contributing factors was even lower, with only 10% recognizing that not completing an antibiotic course leads to resistance, 3.64% identifying that using antibiotics to treat viral infections contributes to the problem, and just 1.3% citing antibiotic sharing as a risk factor. Alarmingly, 15.8% of participants reported not knowing what causes AR. Although the majority (77.47%) of participants recognized AR as a serious issue, 13.47% reported that they did not know, and 9.07% did not consider it a serious problem. These findings are consistent with those observed throughout the study, confirming the existence of knowledge gaps regarding antibiotic use and emphasizing the need for targeted educational interventions.

The results of this study indicate that educational level was the main predictor of AR awareness, with postgraduate degree holders demonstrating significantly higher awareness than those with lower educational levels. These findings align with previous studies that found higher education is associated with greater awareness and improved behavior regarding antibiotic use [24,44,45]. An Italian study reported that dental patients with a higher level of education exhibited better adherence to oral antibiotic therapy [44]. However, local studies involving the general public found no significant link between educational level and knowledge, attitudes, or practices regarding antibiotic use [11,27]. This variation may be attributed to differences in the target population studied.

Several effective targeted educational interventions can be implemented to improve patients’ awareness and understanding of antibiotic use and AR. Global and international organizations recommend the use of patient-centered educational materials, including posters, videos, and leaflets, to educate patients on the proper use of antibiotics and the issue of AR in dental settings. For example, the FDI World Dental Federation recommends using the WHO-produced posters, which contain information on the importance of proper oral hygiene in preventing the development of dental infections and, in turn, reducing the need for antibiotics [46]. Similarly, the Michigan Antibiotic Resistance Reduction Coalition (MARR) encourages the use of posters that cover the benefits and risks of antibiotics used in dentistry, as well as chairside guides on antibiotic use for dental pain, to support antibiotic stewardship in the dental clinic [47]. Active involvement of healthcare professionals, including dentists, in educating their patients is strongly encouraged and plays an essential role in the successful implementation of educational interventions [46,48]

Multiple evidence-based educational interventions implemented in primary care settings have proven effective in improving patients’ knowledge and promoting behavioral change. For example, a study conducted in 20 public health clinics in Malaysia used a face-to-face counseling approach to deliver an educational leaflet on the proper use of antibiotics and the causes of AR. The study found that this intervention effectively improved patients’ knowledge of antibiotic use and AR [49]. Additionally, A US study showed that when patients in clinic waiting rooms were asked to rate statements about the potential harm from unnecessary antibiotics, the statements decreased patients’ likelihood of requesting antibiotics [50]. A UK trial found that educational animated films discouraged patients from requesting antibiotics from general medical and dental practitioners [51]. Also, a local trial showed that gamification, using a board game, improved public knowledge about AMR in relation to dental treatment, demonstrating a better effect than an educational lecture [52].

The present study has several strengths and limitations. The large and geographically diverse sample size provides more generalizable findings and a better representation of the population in Saudi Arabia. Additionally, to our knowledge, this study is one of the first in Saudi Arabia to evaluate knowledge and beliefs about antibiotic use and AR among the public in dental settings. While Alamri et al. investigated adherence behavior among 450 dental patients, our study offers a broader perspective on AR knowledge and misconceptions, as well as the impact of past dental experiences on the perception of antibiotic necessity among dental clinic visitors.

However, this study has specific limitations. The use of an online survey may introduce selection bias as only individuals with internet access and knowledge of how to use social media can complete the survey. Additionally, our sample consisted of a higher percentage of females and participants holding a bachelor’s degree. This demographic distribution may limit the applicability of our findings to the broader Saudi population. Future research should consider stratified sampling or targeted recruitment to ensure demographic representation. Another limitation in our study is the use of self-reported data, which may be affected by response bias, including recall bias and social desirability bias.

Future research should examine adherence behaviors and gain a deeper understanding of patients’ perceptions, expectations, and behaviors regarding antibiotic use in dental settings. We also recommend that future studies evaluate the effect of targeted educational interventions on AR knowledge and antibiotic use within dental settings.

## 5. Conclusions

The current study highlights both the strengths and gaps in awareness of antibiotics and AR among dental clinic visitors. While the majority of participants (70.1%) correctly identified that antibiotics kill bacteria, a proportion reported key misconceptions, particularly about the use of antibiotics for viral infections, the importance of completing the full antibiotic course, and the perceived necessity of antibiotics in dental care. Nearly half of the participants were unaware of the term “antibiotic resistance” and its underlying causes. These findings emphasize the urgent need for enhanced public education and improved communication from healthcare providers. Since previous dental experiences appear to influence patient expectations regarding the necessity of antibiotics, future interventions should focus on enhancing antibiotic stewardship within dental settings and providing targeted patient education to promote responsible antibiotic use and control the spread of AR.

## Figures and Tables

**Table 1:** Sociodemographic characteristics of the respondents (*n* = 1455).

Sociodemographic Characteristics	No. Response (*n*)	Percentage (%)
Gender		
Male	581	39.93
Female	874	60.07
Age (years)		
18–25	636	43.71
26–35	309	21.24
36–45	218	14.98
46–59	210	14.43
>60	78	5.36
NA	4	0.27
Nationality		
Saudi	1397	96.01
Non-Saudi	58	3.99
Education level		
No studies	4	0.27
High school and less	223	15.33
Diploma	165	11.34
Bachelor degree	906	62.27
Postgraduate degree	157	10.79
Province		
Riyadh	685	47.08
Makkah	104	7.15
Medina	115	7.90
Al-Qassim	178	12.23
Eastern	169	11.62
Northern Borders	16	1.10
Tabuk	32	2.20
Al-Bahah	39	2.68
Al-Jawf	59	4.05
Asir	30	2.06
Jazan	3	0.21
Ha’il	2	0.14
Najran	3	0.21
NA	20	1.37

NA: Not Available. This includes four missing responses for age and 20 missing responses for region.

**Table 2:** Prediction of antibiotic resistance awareness by sociodemographic characteristics (*n* = 1431) *.

Sociodemographic Characteristics	N	AOR	95% CI	*p*-Value
Age				
Youth (18–25)	631	1.119	0.8773 to 1.428	0.3658
Adults (26–59)	725	Ref		
Seniors (60+)	75	0.7989	0.4800 to 1.325	0.3845
Gender				
Male	573	0.9621	0.7682 to 1.205	0.7367
Female	858	Ref		
Nationality				
Saudi	1379	Ref		
Non-Saudi	52	1.515	0.8462 to 2.781	0.1684
Region				
Northern	75	1.12	0.6854 to 1.837	0.6513
Western	290	0.9492	0.7173 to 1.256	0.7148
Central	862	Ref		
Eastern	168	1.012	0.7057 to 1.453	0.9475
Southern	36	1.656	0.8335 to 3.424	0.1581
Educational level				
Middle school and less	24	0.8071	0.3400 to 1.851	0.6157
High School	199	0.8145	0.5942 to 1.114	0.2002
Diploma	164	1.003	0.7152 to 1.407	0.9847
Bachelor degree	891	Ref		
Postgraduate degree	153	2.942	1.988 to 4.428	<0.0001

* N = number of respondents. AOR = adjusted odds ratio. CI = confidence interval.

## Data Availability

The original contributions presented in this study are included in the article/Supplementary Material. Further inquiries can be directed to the corresponding author.

## Supplementary Materials

The following supporting information can be downloaded at https://www.mdpi.com/article/10.3390/healthcare13161971/s1: File S1: Survey Questionnaire.
