# Supplementary material for: Knowledge and Misconceptions About Antibiotic Use and Resistance Among Dental Clinic Visitors in Saudi Arabia—A Cross-Sectional Study
Source: Healthcare (Basel). 2025 Aug 11;13(16):1971. doi: 10.3390/healthcare13161971 (PMC12385228; doi:10.3390/healthcare13161971)
Supplement: Supplementary file 1 [file healthcare-13-01971-s001.zip › healthcare-3748340-supplementary.pdf]

Supplementary Material S1: Survey Questionnaire.

Knowledge and Misconceptions About Antibiotic Use and Resistance Among Dental Clinic Visitors in Saudi Arabia **.قياس وعي وإدراك مراجعي عيادة الأسنان باستخدامات المضادات الحيويه**

عزيزي المشارك،

أنت مدعو للمشاركة في هذا الاستبيان، والذي يهدف إلى قياس مستوى معرفة وفهم مراجعي عيادة الأسنان حول المضادات الحيوية ومقاومتها. وبالتالي، يمكن أن يحسن معرفتنا حول مستوى مقاومة المضادات الحيوية والمفاهيم الخاطئة المتعلقة باستخدامها لذا، إذا كنت قد تلقيت العلاج من طبيب أسنان، فهذا الاستبيان موجه لك. لتسهيل مشاركتك، تم تصميم الاستبيان ليكتمل في حوالي 7دقائق . نقدر مشاركتك كثيرًا لأنها ستساعدنا على تحقيق الهدف المنشود من هذه الدراسة. مشاركتك تعتبر طوعية تمامًا. الإجابات ستكون مجهولة الهوية وسرية تمامًا، ولن يتمكن الباحث من تحديد هوية المشاركين. لن يُطلب منك تقديم اسمك أو أي معلومات تعريفية أخرى. سنستخدم هذه المعلومات المجمعة لأغراض البحث فقطز إذا كان لديك أي أسئلة أو استفسارات، فلا تتردد في إرسال بريد إلكتروني إلى الدكتورة إسراء الداوود (ealdawood@ksu.edu.sa) للإجابة على أسئلتك

Dear Participant

You are invited to participate in this survey, and your participation is greatly valued as it will help us to achieve the intended aim of this study. This survey (Perception and Knowledge About Antibiotic Use Among People Who Have Visited a Dental Clinic) aims to measure the level of patient knowledge and understanding about antibiotics and antibiotic resistance. Thus, it can improve our knowledge about the level of antibiotic resistance and misconceptions regarding its use. Therefore, if you have been treated by a dentist, this survey is for you. To make your participation easy, the survey was designed to be completed in approximately 7 minutes. Your participation is completely voluntary. The responses are completely anonymous and confidential, and the researcher will not be able to identify participants. You will not be asked for your name or any other identifying information. We will use this collected information for research purposes only. If you have any questions or concerns, please do not hasitate to email Dr. Esraa Aldawood (ealdawood@ksu.edu.sa) to answer your questions.

Eligibility screening

1. **Are you over 18 years old? هل عمرك يزيد عن 18 عامًا؟**

   O Yes/نعم

   O No/لا

2. **Have you visited a dental clinic in the last five years? هل زرت عيادة أسنان في آخر خمس سنوات؟**

   O Yes/نعم

   O No/لا

Section 1: Demographics

3. **Age (العمر):**

   ____

4. **Gender (الجنس):**

   ○ Male/ذكر

   ○ Female/أنثى

5. **City of Residence (مدينة الإقامه):**

   ____

6. **Nationality (الجنسيه)**

   ○ Saudi (سعودي)

   ○ Non-Saudi (غير سعودي)

7. **Specify the highest educational level achieved (مستوى التعليم)**

   ○ No studies/ غير متعلم

   ○ Primary school/ إبتدائي

   ○ Elementary School/ متوسط

   ○ High School/ ثانوي

   ○ Diploma/دبلوم

   ○ Bachelor degree/ بكالوريوس

   ○ Postgraduate degree/دراسات عليا

   ○ Doctorate degree (PhD)/ دكتوراه

Section 2: Knowledge about antibiotic uses

8. **Which of the following statements about antibiotics do you believe to be true? (Select all that apply) أي من العبارات التالية عن المضادات الحيوية تعتقد أنها صحيحة؟ (اختر كل ما ينطبق)**

- ○ Antibiotics kill bacteria./المضادات الحيوية تقتل البكتيريا.
- ○ Antibiotics kill viruses./المضادات الحيوية تقتل الفيروسات.
- ○ Antibiotics are effective against colds and flu./المضادات الحيوية فعالة ضد نزلات البرد والإنفلونزا.
- ○ It is okay to stop taking antibiotics once you feel better, even if the course is not finished./من الجيد التوقف عن تناول المضادات الحيوية بمجرد الشعور بتحسن حتى لو لم تكتمل الجرعة.

9. **What benefits do you think antibiotics have? You can select more than one option. ما هي الفوائد التي تعتقد أن المضادات الحيوية تقدمها؟ يمكنك اختيار أكثر من خيار واحد.**

- ○ Decrease the pain/ تخفيف الألم
- ○ Decrease inflammation/ تقليل الالتهاب
- ○ Decrease the chance of infection/ يقلل نسبة الإصابة بالالتهاب
- ○ Improve healing/ تحسين التشافي
- ○ No effect/ لا يوجد فائدة
- ○ Do not know/ لا أعلم

10. **What adverse effects do you think antibiotics cause? You can select more than one option. ما هي التأثيرات السلبية التي تعتقد أن المضادات الحيوية تسببها؟ يمكنك اختيار أكثر من خيار واحد.**

- ○ Nausea and/or vomiting/ الغثيان و/أو القيء
- ○ Diarrhea/ الإسهال
- ○ Fever/ الحرارة
- ○ Fungal infection/ عدوى فطرية
- ○ Allergic reaction/ الحساسية
- ○ None of the above/ غير موجود
- ○ Do not know/ لا أعلم

Section 3: The necessity of antibiotics after dental procedures

11. **In the last five years, have you undergone any dental procedures such as teeth extraction, root canal treatment, or gum surgery? في الخمس سنوات الأخيرة، هل خضعت لأي إجراءات طبية للأسنان مثل خلع الأسنان، علاج قناة الجذر، أو جراحة اللثة؟**

○ **Yes/نعم**

○ **No/□**

12. **After undergoing dental procedures such as teeth extraction, root canal treatment, or gum surgery, do you think it is necessary to take antibiotics?**

- بعد الخضوع لإجراءات طبية للأسنان مثل خلع الأسنان، علاج الجذور، أو جراحة اللثة، هل تعتقد أنه من الضروري تناول المضادات الحيوية؟

○ Yes/نعم

○ No /لا

Section 4: Awareness of Antibiotic Resistance

13. **Are you aware of the term "antibiotic resistance"? هل أنت على دراية بمصطلح "مقاومة المضادات الحيوية"؟**

○ Yes/نعم

○ No/لا

14. **If you answered yes, from where did you hear about this term? select all possible answers إذا أجبت بنعم، من أين سمعت بهذا المصطلح؟ اختر جميع الإجابات الممكنة**

○ Doctor/طبيب

○ Nurse/ممرض

○ Pharmacist/صيدلي

○ School curriculum/المنهج المدرسي

○ Friend or relative (including social media)/(صديق أو قريب (بما في ذلك وسائل التواصل الاجتماعي

○ Media (TV, radio, newspapers or other)/(وسائل الإعلام (التلفزيون، الراديو، الصحف أو غيرها

○ Awareness campaign/حملة توعية

○ Other/أخرى

○ I don’t remember/لا أتذكر

15. What do you think causes antibiotic resistance? (Select all that apply) ما الذي تعتقد أنه يسبب مقاومة المضادات الحيوية؟ (اختر كل ما ينطبق)

- O Overuse of antibiotics/الاستخدام المفرط للمضادات الحيوية
- O Not completing the full course of antibiotics/عدم إكمال الجرعة الكاملة من المضادات الحيوية
- O Using antibiotics for viral infections/استخدام المضادات الحيوية للعدوى الفيروسية
- O Sharing antibiotics with others/مشاركة المضادات الحيوية مع الآخرين
- O Other (please specify)/___________________(يرجى التحديد) أخرى
- O I do not know/لا أعلم

16. Do you believe antibiotic resistance is a serious problem? هل تعتقد أن مقاومة المضادات الحيوية مشكلة خطيرة؟

- Yes/نعم
- No/لا
- I do not know/لا أعلم
